# Influence of temperature on methane hydrate formation

**DOI:** 10.1038/s41598-017-08430-y

**Published:** 2017-08-11

**Authors:** Peng Zhang, Qingbai Wu, Cuicui Mu

**Affiliations:** 1 State Key Laboratory of Frozen Soil Engineering, Northwest Institute of Eco-Environment and Resources, Chinese Academy of Sciences, Lanzhou, 73000 China; 2 Key Laboratory of Western China’s Environmental Systems (Ministry of Education), College of Earth and Environmental Sciences, Lanzhou University, Lanzhou, 730000 China

## Abstract

During gas hydrate formation process, a phase transition of liquid water exists naturally, implying that temperature has an important influence on hydrate formation. In this study, methane hydrate was formed within the same media. The experimental system was kept at 1.45, 6.49, and 12.91 °C respectively, and then different pressurization modes were applied in steps. We proposed a new indicator, namely the slope of the gas flow rates against time (*dν*_*g*_ */dt*), to represent the intrinsic driving force for hydrate formation. The driving force was calculated as a fixed value at the different stages of formation, including initial nucleation/growth, secondary nucleation/growth, and decay. The amounts of gas consumed at each stage were also calculated. The results show that the driving force during each stage follows an inverse relation with temperature, whereas the amount of consumed gas is proportional to temperature. This opposite trend indicates that the influences of temperature on the specific formation processes and final amounts of gas contained in hydrate should be considered separately. Our results also suggest that the specific ambient temperature under which hydrate is formed should be taken into consideration, when explaining the formation of different configurations and saturations of gas hydrates in natural reservoirs.

## Introduction

Natural gas hydrates (NGHs) are ice–like crystal compounds consisting of water and natural gas and exist under suitable temperature, pressure, gas saturation, and water salinity conditions, among other factors^1^. In recent decades, extensive exploration via bore holes has demonstrated that NGHs occur widely^2–4^. These hydrate deposits mainly exist in sediments at depths greater than 300 m in the oceans or freshwater lakes, as well as at dozens of meters depth underground in permafrost regions^5, 6^. The total amount of carbon stored in NGH reservoirs in the world is at least twice the total exploitable hydrocarbon reserves stored in the form of traditional fossil fuels^7–9^. Furthermore, these huge NGH reserves play important roles in carbon cycling^10^, climate change^11^, and geological disaster prevention^12–19^.

Laboratory experiments have confirmed all the possible structures of NGHs: Structures I and II^20–22^, Structure H^23^ and a new, yet unnamed one^24^, and some complex mixed structures in natural reservoirs^25, 26^. Moreover, three–phase equilibrium conditions over different temperatures and pressures for different types of hydrates have also been successfully established^27^. Based on these findings, sub-cooling or overpressure relative to the equilibrium conditions have been considered as the sole standard for determining the driving force for hydrate formation.

In field, both vein and nodule hydrate configurations within impermeable mud^28–30^ as well as disseminated hydrates within permeable sand have been observed^31^, and these demonstrate the disparity in hydrate configurations in different geological settings^32^. In other words, factors such as the lithology and sediment matrix, depth of water, and depth at which the gas hydrate is buried might significantly affect the hydrate formation processes^33^. Further, understanding the hydrate formation mechanism is also necessary for the successful application of hydrates in various domains such as natural gas storage and transportation^34, 35^, methane gas extraction from natural gas hydrate reservoirs^36^, and even carbon dioxide sequestration^37^. Unfortunately, so far, a definitive mechanism that can explain well the formation of gas hydrate deposits has not been clarified^38^.

For the specific mechanisms of hydrate nucleation and growth processes, many studies at the molecular scale have been performed using experiment and modeling. Raman spectra of CH_4_–water system indicated that water molecules in the type-I small-cage-like structure surrounded CH_4_ molecules in the solution^39^. Based on a theoretical model, the difference in the chemical potentials of a hydrate building unit in solution and in hydrate crystal was investigated, and then the driving force for crystallization of hydrates was confirmed to be the actual concentration of gas in the solution^40^. Using molecular dynamics (MD) simulations, it was confirmed that guest molecules are dissolved in water, and then multiple guest molecules form amorphous clusters in water-mediated configurations. These amorphous clusters are deemed precursors of nucleation of clathrate hydrates^41^. Their specific formation pathways are significantly affected by the size and solubility of guest molecules^42^, a finding that has been confirmed with macroscopic measurement methods including Raman spectroscopy and X-ray diffraction^43^. Furthermore, using MD simulations, Walsh *et al*.^38^ found that hydrate nucleation occurs only when the concentration of methane gas dissolved in water reaches a certain critical value during the induction period. Subsequently, Guo and Rodger^44^ concluded that while a low temperature is conducive for establishing the water clathrate structure during hydrate formation, a high pressure impedes the formation of this structure.

In order to understand the mechanisms of hydrate formation, many experimental methods have been widely applied ranging from the macroscopic to microscopic scales^45, 46^. Besides the well-known dominating factors for hydrate formation, namely the sub-cooling or overpressure relative to the equilibrium conditions of hydrate, the ambient temperature conditions may also significantly influence the formation processes of hydrates. As pointed out by the geophysical conceptual model from Clennell *et al*.^47^, gas hydrate behaves in a way analogous to ice in a freezing soil, including ‘freeze-drying’ phenomenon and similar disseminated, nodular, layered and massive configurations. However, details of these processes are far from clear.

Since methane hydrate is a representative of NGHs^1^, a series of experiments were designed in this paper to investigate the influence of temperature on the nucleation and subsequent growth of methane hydrate. The results would be helpful for understanding the formation mechanisms of NGHs under natural conditions.

## Results

### Features of the overall hydrate formation process

**Figure 1:**
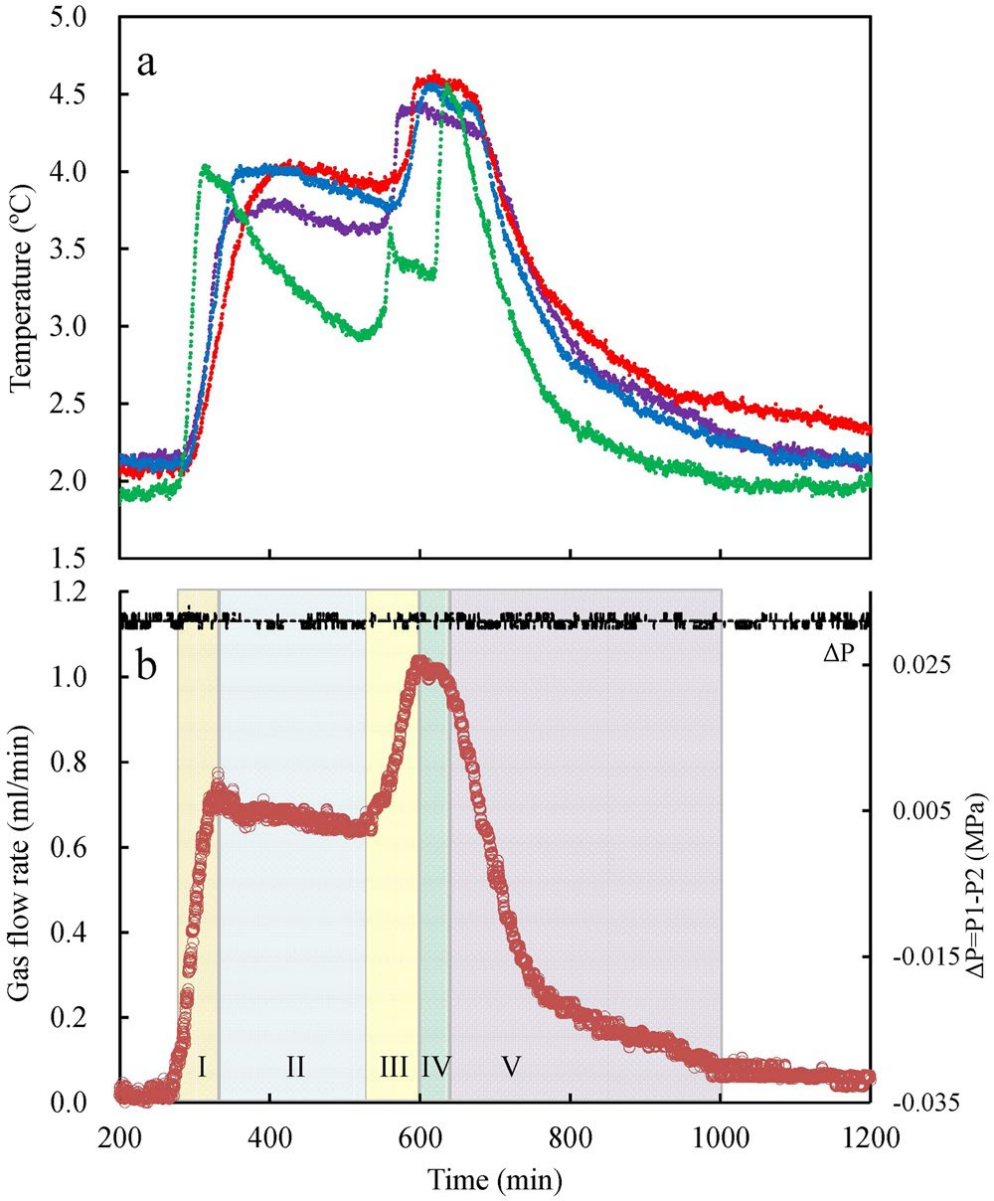
Temperature and gas flow rate during the methane hydrate formation process as a function of time at 1.45 °C. The temperatures at points T1, T2, T3, and T4 are denoted by the purple, red, blue, and green lines (**a**), respectively. The black line in (**b**) shows the pressure difference between P1 and P2.

Three constant temperature conditions were designed to examine the formation process of methane hydrate, namely 1.45, 6.49, and 12.91 °C. For all three temperatures, the changes in pressure, temperature, and gas flow rate at the pump showed similar trends. The results at 1.45 °C are shown in Fig. 1. Because hydrate was formed spontaneously under constant pressure and temperature conditions, changes in the measured time-dependent curves of temperature and gas flow rate indicate the different stages of hydrate formation. From the changing slopes of the temperature and gas flow rate curves (Fig. 1), the formation process can be divided into five distinct stages (stages I, II, III, IV, and V). In stage I, the temperature of the medium rises rapidly and almost simultaneously from the initial value of 2.0 °C to 4.0 °C (Fig. 1a). The gas flow rate also shows a similar increase, rising from the initial value of 0.025 mL/min to 0.716 mL/min (Fig. 1b). Together, these changes indicate that after a certain induction period, numerous hydrate nuclei begin precipitating in the solution within the experimental medium, releasing an appreciable amount of heat. In stage II, the plots of temperature and gas flow rate become comparatively stable, implying that the hydrate crystals continuously form and release heat. Temperature and gas flow rate stability is observed at all points throughout the crystallizer except at the lowest point, T4, which is shown as green line in Fig. 1a. In stage III, a sudden increase in the temperature and gas flow rate is observed. This trend is similar to that in stage I. It indicates secondary nucleation of hydrates, which was also observed by Uchida *et al*.^48^. In stage IV, the changes in the physical parameters are similar to those in stage II, although much shorter in duration of change, and indicate the secondary growth stage of hydrates. In stage V, the temperature and gas flow rate decrease rapidly, indicating the decay stage. In addition, there is almost no pressure difference between P1 and P2 (0.030–0.032 MPa) over the entire formation process.

### Hydrate nucleation process

**Figure 2:**
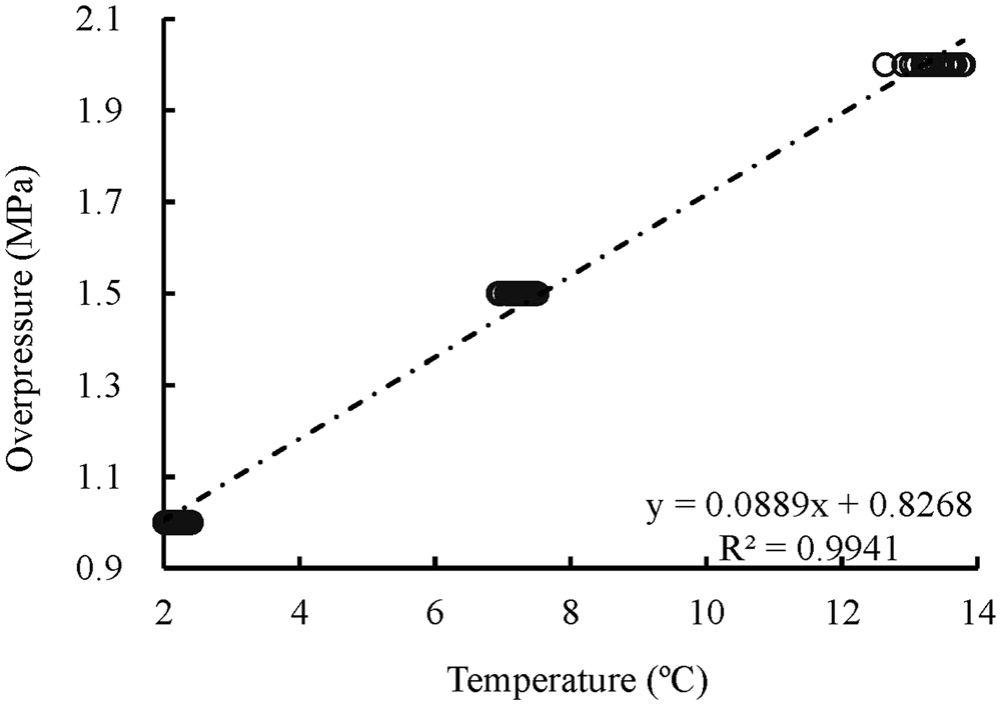
Statistical relationship between the overpressure and temperature during hydrate nucleation. The target pressures of 3, 5, and 10 MPa correspond to the designed temperatures of 1.45, 6.49, and 12.91 °C, respectively.

As listed in Table 1, overpressures of 1.0, 1.5, and 2.0 MPa are required to trigger the hydrate nucleation reactions at 1.45, 6.49, and 12.91 °C, respectively. The actual temperature values measured with each experimental medium are slightly higher than the predesigned temperatures (Table 1). There is a linear relationship between overpressure and measured temperature (Fig. 2).

**Table 1:** Conditions of the initial hydrate nucleation at different locations.

Runs	Measured temperature at different locations under predesigned 1.45 °C (°C)				Nucleation pressure (MPa)	Runs	Measured temperature at different locations under predesigned 6.49 °C (°C)				Nucleation pressure (MPa)	Runs	Measured temperature at different locations under predesigned 12.91 °C (°C)				Nucleation pressure (MPa)
	T1	T2	T3	T4			T1	T2	T3	T4			T1	T2	T3	T4	
3-5-0.5	2.27	2.24	2.18	2.22	4.0	5-5-0.5	7.20	7.21	7.19	7.11	6.5	10-5-0.5	13.79	13.56	13.56	13.40	12.0
3-5-1.0	2.38	2.35	2.38	2.26	4.0	5-5-1.0	7.43	7.36	7.28	6.98	6.5	10-5-1.0	13.66	13.53	13.46	13.26	12.0
3-5-1.5	2.18	2.12	2.05	2.07	4.0	5-5-2.0	7.34	7.30	7.26	6.98	6.5	10-5-2.5	13.19	13.11	12.91	12.63	12.0
3-10-0.5	2.17	2.05	2.12	1.95	4.0	5-10-0.5	7.41	7.38	7.33	6.93	6.5	10-10-0.5	N	N	N	N	12.0
3-10-1.0	2.19	2.15	2.04	1.98	4.0	5-10-1.0	7.43	7.34	7.21	7.10	6.5	10-10-1.0	N	N	N	N	12.0
3-10-1.5	2.06	2.01	2.03	2.02	4.0	5-10-2.0	7.33	7.26	7.18	7.05	6.5	10-10-2.5	13.77	13.77	13.75	13.60	12.0
3-20-0.5	2.12	2.10	2.07	2.06	4.0	5-20-0.5	7.35	7.30	7.12	6.94	6.5	10-20-0.5	N	N	N	N	12.0
3-20-1.0	2.40	2.34	2.24	1.98	4.0	5-20-1.0	7.38	7.14	7.17	7.07	6.5	10-20-1.0	13.77	13.66	13.65	13.56	12.0
3-20-1.5	2.31	2.28	2.12	1.97	4.0	5-20-2.0	7.29	7.24	7.19	6.98	6.5	10-20-2.5	13.21	13.09	13.02	13.03	12.0

N means there was no hydrate nucleation at that location. These pressures are the measured values for the actual nucleation conditions. Some overpressure is required for triggering the hydrate nucleation, and the overpressures of 1.0, 1.5, and 2.0 MPa were measured in this study, corresponding to the designed pressure values of 3.0, 5.0, and 10.0 MPa, respectively.

The data in Table 2 show the stochastic properties of hydrate nucleation^1, 49^. The induction times vary from tens to thousands of minutes without distinct statistical relationship and no relationship with the gas flow rates and pressurization magnitude^50^. However, the statistical results related to the locations at which hydrate nucleation occurred first show a high degree of regularity. Out of the nine experiments at 1.45 °C, in six of the experiments, nucleation occurred first at T1, in one at T3, and in two at T4, as indicated by the numbers (in italics) in Table 2. For the remaining two groups of experiments at 6.49 and 12.91 °C, the statistical results indicate that five occurrences of first nucleation are found at T1, one at T3, three at T4 and one at T1, two at T2, and three at T4, respectively. These results show that the hydrate nuclei tend to form at the T1 location at lower temperatures, as indicated in Table 1. Further, the frequency of occurrence at T1 decreases when the ambient temperature is increased from 1.45 to 6.49 to 12.91 °C, as indicated in Table 2.

**Table 2:** Induction times at four locations within each experimental medium.

Runs	Induction time at different locations (min)				Runs	Induction time at different locations (min)				Runs	Induction time at different locations (min)			
	T1	T2	T3	T4		T1	T2	T3	T4		T1	T2	T3	T4
3-5-0.5	*326*.*67*	339.33	343.33	344.67	5-5-0.5	13.83	10.50	14.33	*6*.*17*	10-5-0.5	881.50	878.50	880.00	*865*.*00*
3-5-1.0	*39*.*00*	48.83	53.33	59.33	5-5-1.0	40.00	40.00	38.00	*30*.*83*	10-5-1.0	5477.83	5387.83	*5366*.*00*	5400.00
3-5-1.5	777.67	775.17	774.00	*771*.*00*	5-5-2.0	*10*.*50*	22.50	28.17	30.17	10-5-2.5	612.167	614.50	*612*.*00*	612.33
3-10-0.5	285.83	285.50	285.17	*280*.*00*	5-10-0.5	*762*.*83*	775.50	778.17	776.17	10-10-0.5	N	N	N	N
3-10-1.0	705.33	709.83	*703*.*50*	713.00	5-10-1.0	*495*.*50*	501.50	496.33	504.00	10-10-1.0	N	N	N	N
3-10-1.5	*2090*.*17*	2108.17	2094.50	2126.83	5-10-2.0	150.17	147.17	*140*.*17*	143.00	10-10-2.5	2795.67	2790.17	2790.67	*2788*.*33*
3-20-0.5	*291*.*17*	301.33	316.00	316.50	5-20-0.5	*2963*.*50*	2971.50	2974.00	2987.00	10-20-0.5	N	N	N	N
3-20-1.0	*1513*.*50*	1516.50	1532.50	1524.67	5-20-1.0	107.50	103.00	100.33	*97*.*33*	10-20-1.0	1159.00	1153.67	1154.33	*1146*.*50*
3-20-1.5	*194*.*83*	208.50	223.83	214.33	5-20-2.0	*256*.*67*	262.00	258.83	265.50	10-20-2.5	*204*.*33*	206.17	205.50	205.50

These italic numbers symbolize the first nucleation occurrences.

### Properties of gas consumption during the various stages

**Figure 3:**
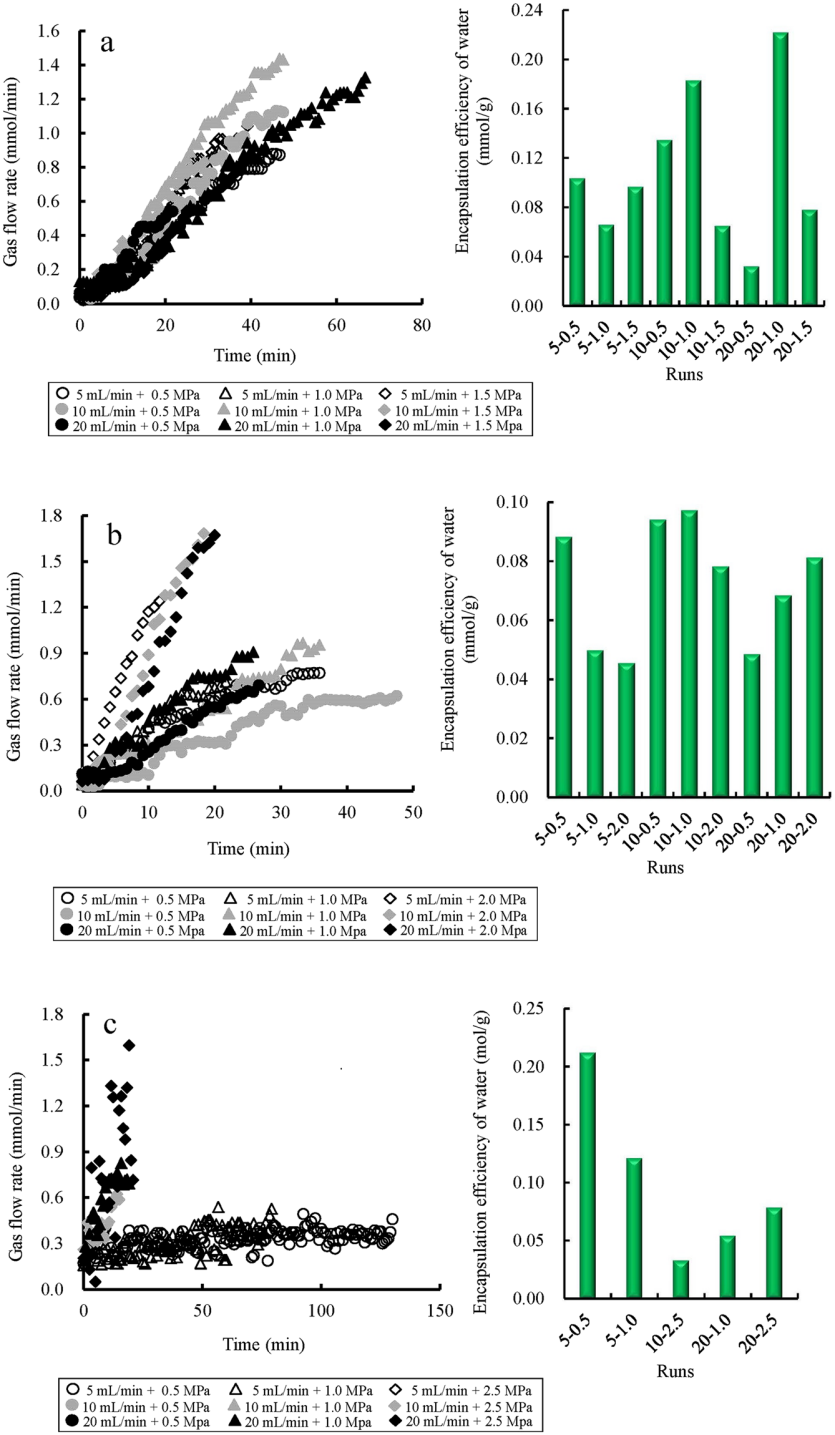
Changes in gas flow rates against time, and final ratios between the amounts of gas contained in the hydrate versus the weight of water during stage I at different predesigned temperatures: (**a**) 1.45, (**b**) 6.49, and (**c**) 12.91 °C.

Changes in the gas flow rates with time during stage I under different temperature conditions are shown in Fig. 3. The slope of each line can be read directly from Fig. 3. Since these lines represent the changes in gas flow rates against time (*dν*_*g*_ */dt*), the unit of their slopes is mmol/min^2^. The total amount of methane gas consumed for hydrate formation during this stage was also calculated. The average values of the *dν*_*g*_/*dt* and the normal amounts of consumed gas are 0.015 mmol/min^2^ and 0.11 mmol/g at 1.45 °C, 0.016 mmol/min^2^ and 0.072 mmol/g at 6.49 °C, and 0.0031 mmol/min^2^ and 0.099 mmol/g at 12.91 °C, respectively. Therefore, the maximum of the *dν*_*g*_/*dt* occurs at the middle temperature, whereas the maximum of gas amount occurs at the lowest temperature.

When not considering the exceptions indicated by the diamond data markers in Fig. 3b and the all-solid and gray markers in Fig. 3c, the slopes of the lines at the lowest temperature are distinctly higher than those at other temperatures: 0.015 mmol/min^2^ at 1.45 °C, 0.0085 mmol/min^2^ at 6.49 °C, and 0.0003 mmol/min^2^ at 12.91 °C. On the contrary, the normal amount of gas consumed is greatest at the highest temperature: 0.166 mmol/g at 12.91 °C, 0.11 mmol/g at 1.45 °C, and 0.074 mmol/g at 6.49 °C. The different data markers represent different pressurization modes.

The exceptions noted in Fig. 3b imply that all the modes at the greatest (2.0 MPa) magnitude per pressurization step significantly increase the line slopes (*dν*_*g*_/*dt*). However, at the highest temperature of 12.91 °C, similar effects are observed at the higher pressurization rate of 20 mL/min. On the other hand, the line slopes at the lowest temperature, 1.45 °C are barely affected by the two above pressurization modes. As shown in Fig. 3a, the lines almost converge, and show no significant differences among their slopes. The missing lines in Fig. 3c suggest that there were no corresponding stages during the formation processes. These missing lines are frequently found in the subsequent stages of formation.

**Figure 4:**
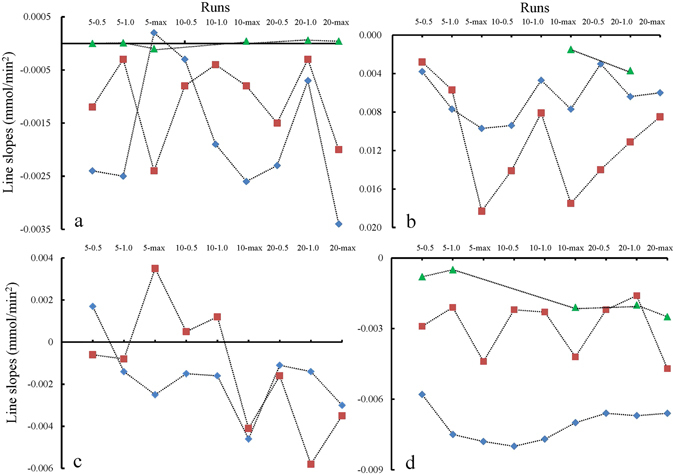
Slopes in the plots of gas flow rates versus time over stages II–V, denoted as a–d respectively. The diamond, square, and triangle markers represent the experiments at 1.45, 6.49, and 12.91 °C, respectively.

**Figure 5:**
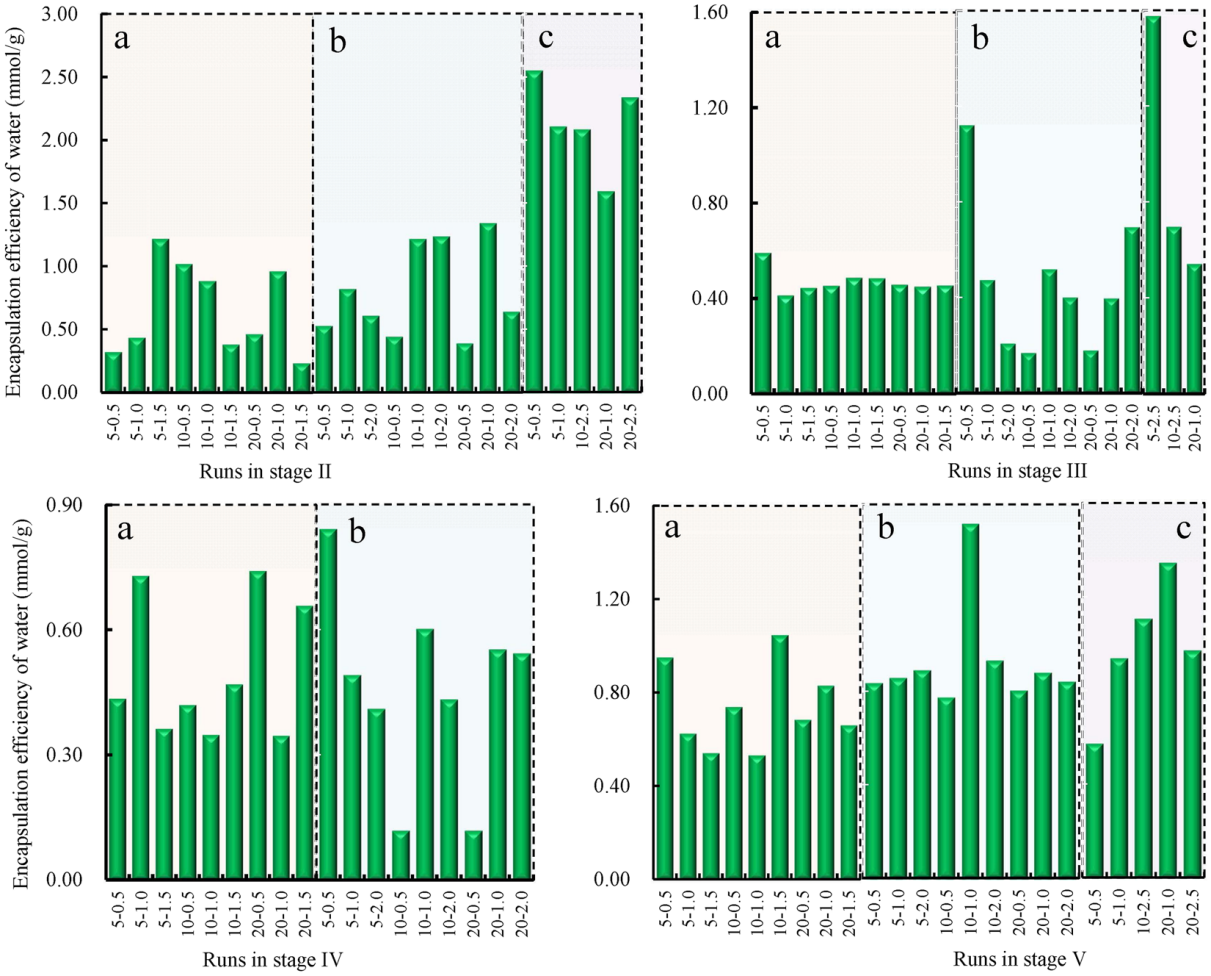
The normal amounts of gas contained in hydrate over stage II–V. Labels (**a**,**b** and **c**) corresponds to the experiments at 1.45, 6.49, and 12.91 °C, respectively.

Using the same method as that used in stage I, the line slopes (*dν*_*g*_/*dt*) and normal gas amounts over stage II were also obtained. As shown in Fig. 4a, unlike stage I, most of the line slopes in stage II are negative and their magnitudes tend to increase with increase in temperature. The average values of *dν*_*g*_/*dt* are −0.0018 mmol/min^2^, −0.0011 mmol/min^2^, and 8 × 10^−6^ mmol/min^2^ at 1.45 °C, 6.49 °C, and 12.91 °C, respectively. The pressurization mode does not have any obvious systematic effect on the line slopes. Further, unlike stage I, the averages of the normal amounts of gas consumed by hydrate increase continuously with increasing temperature as shown by the stage II in Fig. 5, i.e., 0.65 mmol/g, 0.80 mmol/g, and 2.13 mmol/g at 1.45 °C, 6.49 °C, and 12.91 °C, respectively. Obviously, these two trends are contrary to each other.

Unexpectedly, the secondary methane hydrate nucleation phenomena occurred spontaneously rather than at the completion of the growth stage, as shown in Fig. 1. As evident from Fig. 4b, all the line slopes (*dν*_*g*_ */dt*) were positive, with the highest average value of 0.011 mmol/min^2^ at 6.49 °C, followed by 0.0065 mmol/min^2^ at 1.45 °C and 0.0026 mmol/min^2^ at 12.91 °C. On the contrary, as shown by the stage III in Fig. 5, the average normal gas amount of 0.94 mmol/g was highest at 12.91 °C, followed by 0.47 mmol/g at 1.45 °C and 0.46 mmol/g at 6.49 °C.

A secondary growth stage was observed for methane hydrate after secondary nucleation. Similar to stage II shown in Fig. 4a, most of the line slopes (*dν*_*g*_ */dt*) for the plots of gas flow rate against time turn negative again (Fig. 4 c). As shown by stage IV in Fig. 4, the average values are almost the same as those in stage II: −0.0017 mmol/min^2^ at 1.45 °C and −0.0012 mmol/min^2^ at 6.49 °C. Additionally, the average of the normal amounts of gas consumed for hydrate formation at 1.45 °C is 0.50 mmol/g, which is slightly higher than that of 0.46 mmol/g at 6.49 °C. In addition, Fig. 4c also shows that at the highest temperature of 12.91°C, all the secondary growth stages did not occur.

After the secondary growth, the entire hydrate formation process tended to end and underwent the last decay stage (Fig. 1). Figure 4d shows that all the slopes of the plots of gas flow rates versus time over this decay stage change to negative values. The line slopes (*dν*_*g*_/*dt*) at 12.91 °C have the largest magnitudes with an average of −0.0016 mmol/min^2^, followed by those at 6.49 °C with an average of −0.0030 mmol/min^2^, and those at 1.45 °C with an average of −0.0071 mmol/min^2^. As shown by the stage V in Fig. 5, in the case of the normal gas amounts contained in the hydrate, the values at 12.91 °C are the largest with an average of 0.98 mmol/g, followed by those at 6.49 °C, and those at 1.45 °C in order, with averages of 0.92 mmol/g and 0.73 mmol/g, respectively.

**Figure 6:**
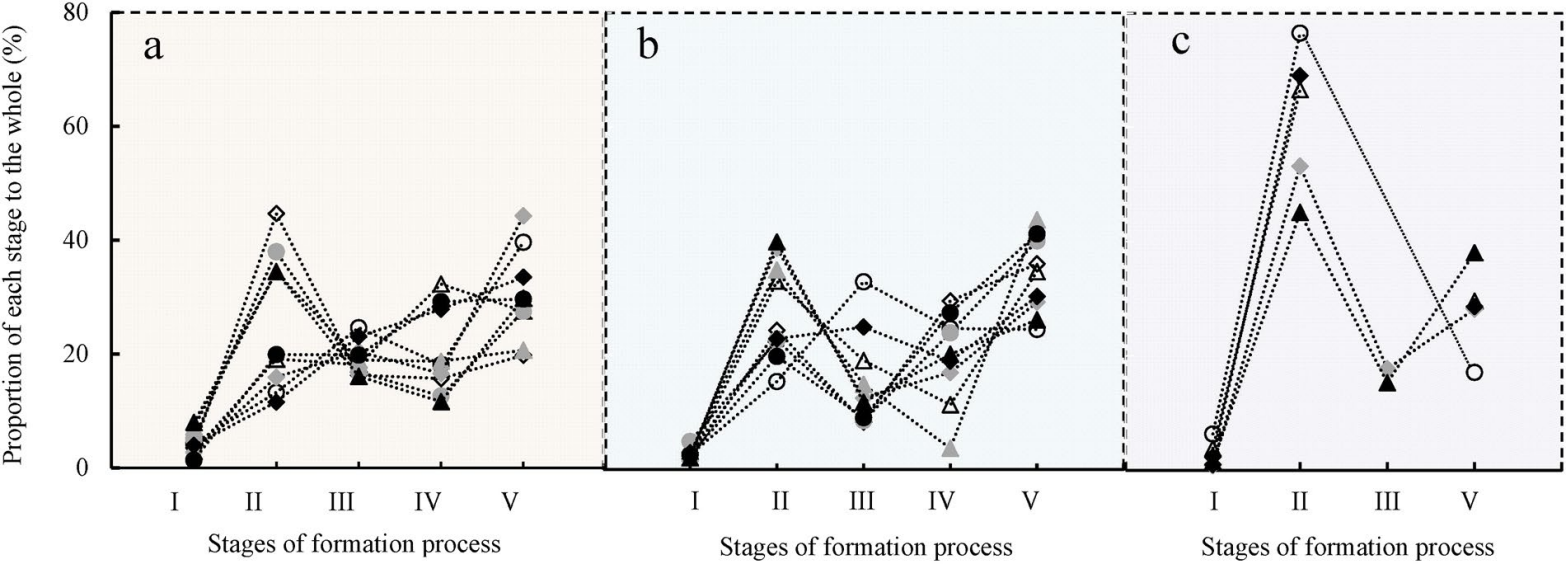
Ratio of the amount of gas consumed in each stage to that consumed during the whole process.

The ratios of amounts of gas consumed during each stage to those consumed during the entire formation process were calculated for each experiment. As shown in Fig. 6, the gas consumption ratios for the hydrate nucleation (expressed as percentage) at 1.45 °C are the largest with an average value of 4.33%. At 6.49 °C and 12.91 °C, the gas consumption ratios are 2.67% and 2.97%, respectively. At the lower temperatures of 1.45 °C and 6.49 °C, the highest amounts of gas are consumed in stage V, with averages of 30.27 and 33.99% respectively, followed by stages II, IV, and III, with average values of 25.71, 20.32, and 19.37% at 1.45 °C and 27.94, 19.63, and 15.77% at 6.49 °C, respectively. However, at 12.91 °C, the highest gas consumption periods all occur in stage II, with an average value of 62.09%, followed by stages IV and III with average values of 28.32 and 16.54%, respectively.

### Hydration number after each process

**Figure 7:**
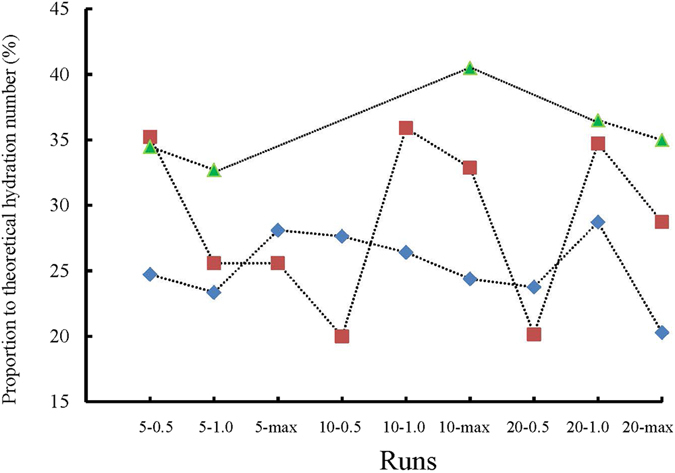
Ratio between the final amount of gas enclosed in the hydrate and the theoretical value for each experiment. The diamond, square, and triangle markers represent the experiments conducted at 1.45, 6.49, and 12.91 °C, respectively.

The hydration numbers obtained after each experiment were also calculated, and then the ratios of the calculated to theoretical hydration number (5.75)^14^ were obtained. The results are plotted in Fig. 7. As shown in Fig. 7, the final amounts of methane gas encapsulated in the hydrate over the entire course of experiments are very small, with the highest hydration number only reaching 40.51% of the theoretical value of 5.75. The hydration numbers tend to decrease with increase in temperature and the ratios of experimental to theoretical hydration numbers increase. The average proportions to the theoretical hydration numbers are 25.26, 28.75, and 35.84% at 1.45, 6.49, and 12.91 °C, respectively.

## Discussion

In this study, the crystallizer was cooled by air blowing upwards from the base. The temperatures at the top location (T1) are all slightly higher than those at the bottom location (T4). The methane hydrate tends to nucleate first at the higher temperature location T1 (Table 2). At 1.45 °C, in six out of nine experiments, the hydrate was found to first nucleate at T1, whereas at 6.49 °C, this nucleation phenomenon occurred in five out of nine experiments. It has been confirmed that besides the promotion of hydrate formation by the silica surfaces^51^, the concentration of methane gas in water is a dominating factor for hydrate nucleation in both pure water and porous medium systems^38, 39, 52^. Owing to the higher temperature at T1, the methane gas diffuses into liquid water and reaches a critical concentration at a higher rate at this location, which then leads to faster hydrate precipitation. However, this fact contradicts the results calculated with the driving force based on thermodynamic states.

Sub-cooling or overpressure is defined as the difference between the theoretically calculated equilibrium conditions and measured ones and has been traditionally regarded as being the determinant for the driving force of hydrate formation. Our results show that all the locations within each experimental medium were under the same stable pressure during hydrate nucleation (Table 1). Theoretical equilibrium temperatures of methane hydrate corresponding to pressure values of 4.0 MPa and 6.5 MPa were calculated as 4.31 °C and 9.00 °C, respectively with the CSMGem software. Further, the difference in pressure between the top and bottom ends of the crystallizer (ΔP) in Fig. 1 shows that the methane gas disseminated uniformly throughout the experiment. Therefore, the lower temperature location T4 has a greater driving force for hydrate formation. Conversely, hydrate nucleation at 1.45 °C and 6.49 °C were first measured at location T1 which was under slightly higher temperatures.

At the highest temperature of 12.91 °C, this trend was broken and three out of the nine nucleation occurrences were first detected at T4 (Table 2). This is mainly attributed to the following reasons. Before the concentration of methane gas in solution reaches the critical value at which nucleation is triggered^33^, the solution must experience the supersaturated state first. In this state, the solubility limit of methane gas in the solution is exceeded, which is accomplished by the formation of incomplete clathrate structures of water molecules^44^. According to the conclusions of Guo and Rodger^44^, lower temperature is conducive to the formation of these clathrate structures. As a result, first nucleation of hydrate occurs preferentially at the low temperature location T4 at 12.91 °C. On the other hand, the results from Guo and Rodger^44^ also imply that a higher ambient temperature impedes the formation of the water clathrate structures. This is also verified in the present study. According to the data presented in Tables 1 and 2, nucleation did not occur in the three experiments conducted at the highest temperature of 12.91 °C, even when an induction time much longer than those used for other experiments in the same group was provided.

Our results suggest that for hydrate nucleation, lowering the temperature is more effective than the formation driving force derived from the equilibrium thermodynamic states, while the hydrate exists stably. In addition, the pressurization modes do not show any influence on hydrate nucleation. During the hydrate growth process, the cooling air being blown upwards can undoubtedly induce a vertical temperature gradient within the experimental medium. This may further cause heterogeneous formation of hydrate within the same medium. In this study, the formation processes of hydrate are represented by the changing amounts of consumed methane gas under different temperature conditions. As a result, the effects of heterogeneous hydrate formation caused by temperature gradient could be neglected. In addition, before the pressurization procedures, the experiment system was left undisturbed overnight under the pressure condition 0.5 MPa lower than the target value. During that period, the temperature within the sample in the radial direction would become homogeneous. Because the samples used in this study were all unsaturated, methane gas could diffuse throughout the whole sample rapidly while being pressurized. As a result, the radial temperature gradient should also have little influence on hydrate formation.

In this study, all experiments used the medium of silica gel powder with a water content ratio of 1.5:1 (*W*_*water*_ */W*_*media*_) and a height of 14 cm. Because the stability of methane hydrates is extremely susceptible to changes in environmental conditions^53^, the methane hydrates were all formed under constant temperature and pressure conditions. Using the same medium could exclude the complex effects of the medium property on the formation processes of gas hydrate, including grain size^54^, material and character of the surface^55, 56^, mineral composition^57^, salinity^58^, clay content^59, 60^, and volume of porous media^61^. The media were also designed to be unsaturated in order to provide a large gas-water contact surface of fixed area in each experiment. Finally, the influences of temperature on the nucleation and subsequent growth processes of methane hydrate were investigated. Therefore, it is possible to explain our results using some conclusions from the MD simulations.

Since methane hydrate is formed under stable pressure and temperature conditions, the driving force for formation can be represented with the line slopes of the plots of gas flow rate versus time (*dν*_*g*_/*dt*) in units of mmol/min^2^. Unlike the traditional driving force derived from equilibrium thermodynamic states, these line slopes actually indicate acceleration in gas flow rates and represent the intrinsic driving force from the view of materials (flow rates of gas amounts).

During the initial nucleation stage, the driving forces decrease upon increase in temperature, without considering the influence of pressurization mode. Conversely, the normal amounts of gas contained in the hydrate over this stage increase overall following the increase in temperature. Further, the pressurization modes exert some influence on the driving force. The driving force at 6.49 °C was significantly enhanced at the pressurization rate of 1.5 mL/min, and those at 12.91 °C were enhanced at pressurization magnitudes of 1.0 MPa and 2.5 MPa.

During the initial growth stage, the slopes of the major lines turned negative, implying that the hydrate growth is actually a decelerating process at this stage. The absolute value of line slope represents the magnitude of the intrinsic driving force. In conclusion, the magnitude of driving force over this growth stage still decreases upon increase in temperature. Conversely, the normal amounts of gas contained in the hydrate also increase with increase in temperature. The calculation results indicate that the hydrate growths at 1.45 °C and 6.49 °C are actually decelerating processes, whereas that at 12.91 °C is a slow accelerating process.

Similar to that observed by Uchida *et al*.^48^, the secondary nucleation phenomenon was also distinctly measured in this study. The occurrence of the secondary nucleation phenomenon might be attributed to the following reasons. After the initial nucleation, methane hydrate begins to grow owing to sufficient gas supply under a constant pressure. The fact that the abundant point defects are formed over the spontaneously formed hydrate structures has been confirmed^44^. These defective structures are different from the strictly regular type I structure of methane hydrate, which is obtained only after many formation–dissociation cycles and has a hydrate number of almost 5.75^14^. Therefore, the internal potential of the hydrate system with the regular crystal structure reaches the minimum value. On the contrary, the methane hydrate formed by a single spontaneous formation process cannot occur with this regular structure. Many unstable pseudo-cages will then appear over this single spontaneous formation process, which are generally empty or occupied by water molecules^44^. As a result, enormous crystal point defects are formed in the hydrate structure, which are primarily caused by defect stabilization arising in the absence of H-bonding between water and the guest molecules^62^. Following hydrate growth, these defects accumulate continuously and the internal potential of the hydrate system increases constantly. As a result, the potential of the gas solution surrounding the hydrate system is also enhanced. If this was not the case, the hydrate would be expected to stop growing and begin dissociating. Finally, the gas solubility in the liquid water surrounding the hydrate is continuously enhanced for maintaining continuous hydrate growth, which makes secondary nucleation possible.

In the secondary nucleation stage, the slopes of all the plots of gas flow rate versus time are positive, implying this stage is also an accelerating process during hydrate growth. However, unlike the initial nucleation stage, the magnitude of driving force at 6.49 °C is larger than that at 1.45 °C, whereas that at 12.91 °C is the smallest. This difference implies that besides temperature, details of the hydrate structures formed during the growth stage might also be an important factor affecting the magnitude of driving force during secondary hydrate nucleation. Furthermore, the normal gas amounts are also inversely related to the magnitude of driving force.

In the secondary growth stage, hydrate formation is also a decelerating process and similar to stage II, the extent of deceleration decreases with increase in temperature. The average of the normal amount of gas contained in the hydrate at 1.45 °C is slightly higher than that at 6.49 °C. This is mainly due to the fact that higher temperature attenuates the secondary growth process of the hydrate. As a result, there is no secondary growth stage at the high temperature of 12.91 °C. Therefore, the much shorter period of secondary growth at the high temperature compared to that at the low temperature implies that much less amount of gas is consumed during hydrate growth in the former case.

In the final decay stage, the hydrate formation tends to end and this stage is also a decelerating process. Compared to the two decelerating growth processes, namely the initial and secondary growth processes, the extent of deceleration in hydrate formation during this stage is higher and also decreases with increase in temperature. The normal amounts of gas contained in the hydrate during this stage are also inversely related to the magnitude of driving force. This decay stage may represent an annealing process of the hydrate crystalline^63^. Over this stage, the crystal structure of the hydrate formed in the medium becomes more regular, the internal energy is further reduced, and therefore the whole hydrate system becomes more stable. This stage might be mainly a self-regulation process of the hydrate crystalline when the whole hydrate formation process is close to completion^63^. As a result, the rates of gas consumption and heat release both slow down gradually during this stage (Fig. 1).

**Figure 8:**
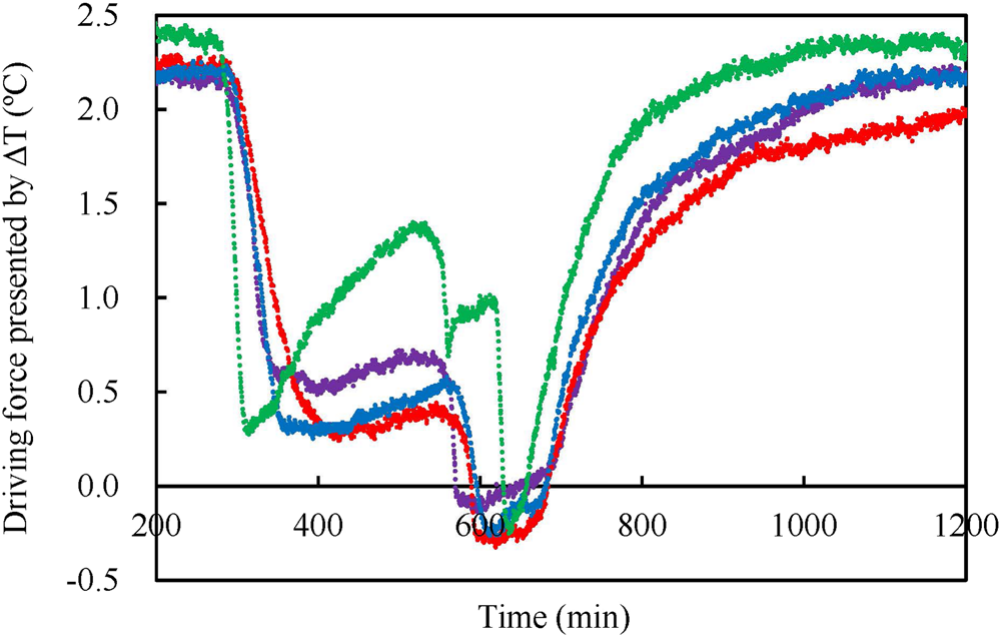
Driving force indicated by the sub-cooling temperature, which was calculated as the difference between the theoretical equilibrium temperature of 4.31 °C at 4.0 MPa and the temperature measured during the experiments shown in Fig. 1.

Based on the above analyses, it is evident that the magnitudes of driving force presented from the view of material (flow rates of gas amounts) are completely different from those calculated traditionally using the equilibrium hydrate formation conditions. The traditional driving forces shown in Fig. 8 suggest dynamic changes during the growth process. Under the stable pressure of 4.0 MPa applied in this study, the phase equilibrium temperature of methane hydrate is calculated to be 4.31 °C, using the CSMGem software. Therefore, the driving forces calculated with the differences between 4.31 °C and the measured temperatures signify real-time changes and the driving forces are determined from the measured values. However, the driving forces calculated using the changes in the gas flow rates show a fixed value over each formation stage because based on the changes in the real amounts of methane gas. It could be inferred that the latter are the intrinsic driving forces for methane hydrate formation. Our results show that over each formation stage, this intrinsic driving force is fixed and its magnitude is inversely related to measured temperature. This implies that the magnitude of driving force for the spontaneous hydrate formation process occurring at a lower temperature is greater than that at higher temperature. On the contrary, the traditional driving force expressed by sub-cooling or overpressure presents no direct relation with the pure temperature conditions^64^. Our results also show that the final amounts of methane gas contained in the hydrate are generally contrary to the magnitudes of driving force. Therefore, it could be concluded that the temperature at which methane hydrate is formed can also affect the efficiency of encapsulation of gas by the hydrate. In other words, a low temperature implies low encapsulation efficiency.

In the case of pressurization modes, except for a few obvious influences exerted by them on the magnitude of driving force over the initial nucleation stage, there is no obvious influence over the other stages of formation. In the spontaneously formed methane hydrate, its final amount of gas content is very low, with the maximum being only 40.51% of the theoretical hydration number of 5.75. This value is significantly lower than 57.5% achieved in the pure silica sand bed with 75% water saturation^59^. This could be explained by that the small particle size in this study lowers the amount of gas consumed by methane hydrate^60, 65^.

Since low temperature can enhance the magnitude of driving force for hydrate formation, the ratios of the amount of gas used for initial nucleation to the total amount consumed over the entire process is the greatest at 1.45 °C (shown in Fig. 6). At the highest temperature of 12.91°C, methane gas is mainly consumed during the initial growth stage, i.e., 45.10–76.51% of the total gas amount is consumed. This ratio is much higher than that consumed over the other stages. MD simulations confirmed that low temperature is conducive for the establishment of the water clathrate structure during the methane hydrate formation process. This structure will include some unstable pseudo-cages, which are either empty or occupied by water molecules^44^. Therefore, it is reasonable that the methane hydrate formed under the highest temperature in this study should have the most regular clathrate structure. Accordingly, only two secondary nucleation occurrences and no secondary growth are observed during the hydrate formation process at 12.91 °C.

Taken together, our results show that the influence of temperature on the hydrate saturation within sediments should be considered carefully, in order to accurately estimate the real natural gas hydrate resources on Earth.

## Methods

### Materials and experimental apparatus

We chose silica gel powder (Yucheng Chemical Co., Ltd., Shanghai, China) as the experimental medium to represent the natural deposits because it has a fixed water-gas contact surface area at a given water content. The medium has a density of 0.35 g/cm^3^, porosity of 77.44% and an average particle size between 25 and 58 µm. When saturated, the water content: medium ratio is 2.2:1 (*W*_*water*_ */W*_*media*_). The purity of methane gas was 99.99% (Yongfang Chemical Co., Ltd., Lanzhou, China).

**Figure 9:**
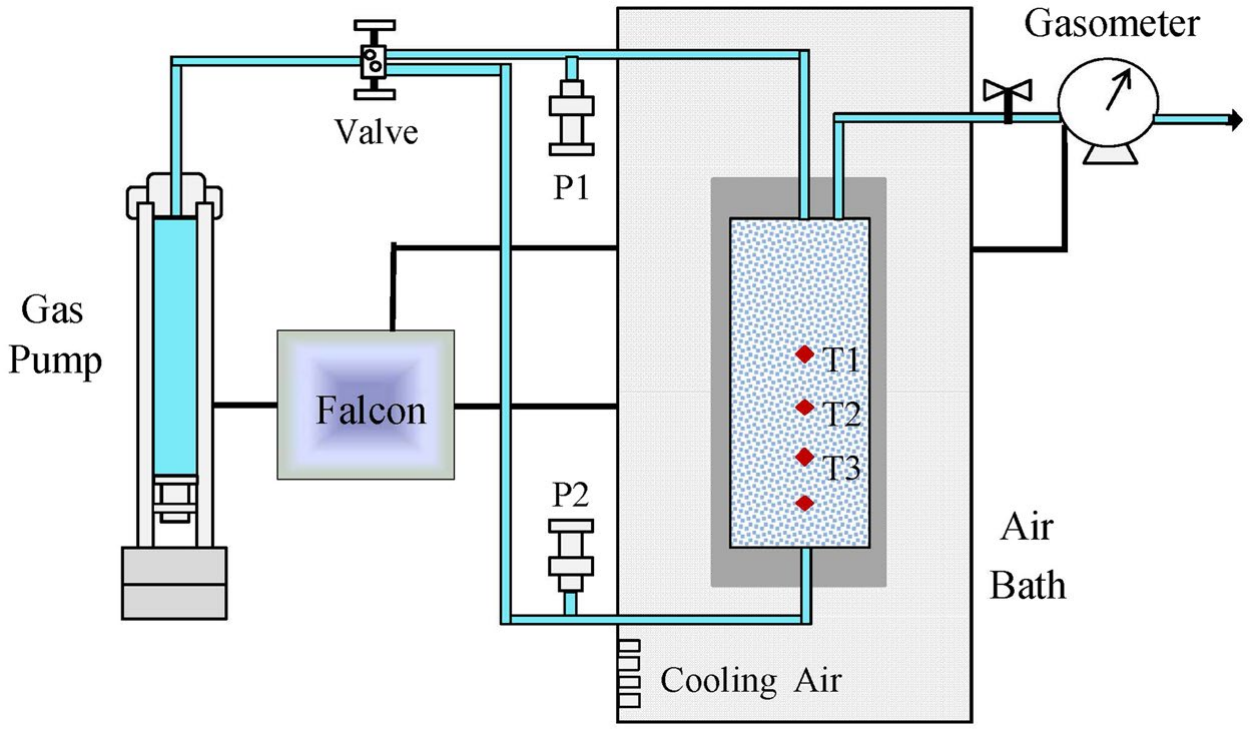
Schematic diagram of the experimental apparatus.

The experimental system was custom–designed and assembled by Sanchez Technologies, France (Fig. 9). The system consists of a digitally controlled gas pump, control software (Falcon), and crystallizer (with a height of 14 cm, diameter of 6.2 cm, without stirring function) fixed in an air bath (80 cm × 50 cm × 80 cm). The temperature of the crystallizer was regulated with an air bath, in which cooling air was blown continuously upwards from the base. The device contains four temperature sensors (−20 to 50 °C with a resolution of 0.01 °C) at different heights in the crystallizer labelled T1, T2, T3, and T4 at 7.0, 5.1, 3.1, and 1.1 cm, respectively. Additionally, there are also two pressure sensors P1 and P2 (0–50 MPa with a resolution of 0.001 MPa) connected to the bottom and top ends through stainless steel conduits, respectively.

The experimental media with fixed water content of 1.5:1 (*W*_*water*_ */W*_*media*_), which appear unsaturated, were used through all experiments. This water content leads to a large gas–water contact area. It also provides the highest water conversion ratio for hydrate formation^66, 67^, as well as more interconnected pore space which facilitates the formation of hydrate throughout the experimental medium^68^.

### Experimental procedure

Before each experiment, the mixed medium of silica gel powder and ultra–pure water with 1.5:1 water content (*W*_*water*_ */W*_*media*_) was charged into the crystallizer, until the height of the medium reached 14 cm. Therefore, the weight of liquid water finally charged into the crystallizer was 200 ± 20 g. The whole system was then purged for 20 min using 15 L of methane gas at atmospheric pressure, as measured using a gasometer. The temperature of the air bath was then rapidly reduced to the calculated target value, after which it was maintained constant. The gas pressure was increased to an initial preparatory value that was 0.5 MPa lower than the target pressure. Finally, the whole system was left undisturbed overnight to allow the methane to dissolve sufficiently.

In order to simulate the formation conditions of natural NGH reservoirs, the temperatures were first maintained at constant values and the pressure increased in stages. Subsequently, the gas pressure was regulated using a gas pump to promote methane hydrate formation. Three target pressure values, namely 3, 5, and 10 MPa were chosen and the equilibrium temperatures were calculated as 1.45, 6.49, and 12.91 °C, using CSMGem (Natural Gas Hydrate Center, Colorado School of Mines).

In order to examine the influence of gas charging patterns on hydrate formation, three different flow rates (5, 10, and 20 mL/min) combined with three pressurization magnitudes (0.5, 1.0, and 1.5 or 2.0 or 2.5 MPa) were considered. The “1.5 or 2.0 or 2.5 MPa” actually represent the same pressurization procedure that was carried out from the initial condition (0.5 MPa lower than the target pressure) to the practical one in a single step. The practical pressures under the designed temperatures of 1.45, 6.49, and 12.91 °C were respectively confirmed as 4.0, 6.5, and 12.0 MPa, as listed in Table 1. For example, for the practical pressure of 12.0 MPa, an additional pressure of 2.5 MPa is required for the one-step pressurization procedure, namely from 9.5 MPa (0.5 MPa lower than the target 10 MPa) to the practical 12.0 MPa. Obviously, this pressurization mode was performed only in the last one in each group of experiments.

Under each designed temperature condition, all combinations of flow rate and pressure magnitude will be tested. For example, the experiment at 1.45 °C was conducted as follows. The temperature was firstly decreased to 1.45 °C, and then the pressure was continuously increased to 2.5 MPa (0.5 MPa lower than the target 3.0 MPa) at a certain rate. The whole system was then left undisturbed overnight. Subsequently, gas was added at a rate of 5 mL/min until the pressure reached the target of 3.0 MPa. The system was then left undisturbed for over 12 h. If hydrate nucleation (as detected via a sudden temperature rise) was not observed over that period, the same pressurization procedure (i.e. 5 mL/min flow rate and 0.5 MPa magnitude) would be repeated, followed by another >12 h of equilibration to provide sufficient induction time for hydrate nucleation. Once the pressurization procedure was completed, the “constant pressure” function of the gas pump was immediately turned on. As a result, hydrate could nucleate and grow spontaneously under a fixed pressure value with a continuous, sufficient gas supply. After this experiment was completed, the next one was conducted at the same gas flow rate, only the pressurization magnitude was changed from 0.5 to 1.0 MPa. Thus, 9 combinations of flow rate and pressurization magnitude were implemented under each temperature condition, adding to a total of 27 experiments as listed in Table 1.

Since the nucleation of hydrates is difficult to predict, the pressure was increased stepwise in order to determine the nucleation conditions. The interval between two pressurization steps was set to be over 12 h to allow sufficient induction time for nucleation. In other words, the system was equilibrated again before the hydrate nucleated. For each temperature condition, the various pressurization modes consisting of different gas flow rates and pressure values were examined. As a result, nine valid experiments were conducted under each temperature condition, as listed in Table 1.

### Calculation methods

For calculating the induction time for nucleation in this study, the zero-time point was defined as the time at which the last pressurization operation before hydrate nucleation was completed. During each experiment, all the physical parameters were recorded and saved by the Falcon software at intervals of 10 s. The data output by the gas pump was in units of mL/min. The amounts of inflowing gas were calculated using the real gas equation *PV* = *nZRT*. The compressibility factor, *Z*, was calculated using the Pitzer correlation^69, 70^. Slight changes in *Z* during the same experiment were neglected. The normal amounts of gas consumed by hydrate formation were calculated in terms of unit weight of water in each sample. The gas flow rates were also converted into units of mmol/min using the gas equation. Additionally, the slopes of the plots of gas flow rates against time (*dν*_*g*_/*dt*) were easily obtained using Microsoft Excel and in units of mmol/min^2^.

The different experimental runs were named using the following convention: pre–designed pressure value (MPa) – gas flow rate (mL/min) – magnitude per pressurization operation (MPa). The temperatures of the experiments were pre-designated at 1.45, 6.49, or 12.91 °C.

## Conclusions

The influence of temperature on the methane hydrate formation was investigated via experiments on the spontaneous formation of hydrate under different stable temperature and pressure conditions. Methane hydrate was formed with the pressurization method, in which methane gas was pressurized to certain values with different modes and the methane gas was then constantly supplied by an automatic gas pump. The slopes of the gas flow rates against time (*dν*_*g*_ */dt*, in a unit of mmol/min^2^) and the amounts of gas consumed by hydrate formation were calculated. These slopes represent the acceleration in gas flow rates, and therefore they indicate the magnitude of driving force for hydrate formation. The results show that there are five separate stages in the whole spontaneous formation process of methane hydrate, namely the initial nucleation and growth, secondary nucleation and growth, and decay. Except the obvious influence of pressurization mode on the magnitudes of driving force during the initial nucleation stage, there are no significant influences during the other stages. The magnitudes of driving force are generally inversely correlated to the temperature at which the hydrate is formed. Conversely, the amounts of gas consumed by hydrate during each stage are proportional to the temperature. The results imply that the specific temperature conditions under which hydrate was formed should be carefully considered, when explaining the formation of different configurations and saturations of gas hydrates in natural reservoirs.
